# Multi-line ssGBLUP evaluation using preselected markers from whole-genome sequence data in pigs

**DOI:** 10.3389/fgene.2023.1163626

**Published:** 2023-05-12

**Authors:** Sungbong Jang, Roger Ros-Freixedes, John M. Hickey, Ching-Yi Chen, William O. Herring, Justin Holl, Ignacy Misztal, Daniela Lourenco

**Affiliations:** ^1^ Department of Animal and Dairy Science, University of Georgia, Athens, GA, United States; ^2^ Departament de Ciència Animal, Universitat de Lleida-Agrotecnio-CERCA Center, Lleida, Spain; ^3^ The Roslin Institute and Royal (Dick) School of Veterinary Studies, The University of Edinburgh, Edinburgh, Scotland, United Kingdom; ^4^ The Pig Improvement Company, Genus plc, Hendersonville, TN, United States

**Keywords:** ssGBLUP, whole-genome sequence data, marker preselection, multi-line evaluation, unknown parent groups, metafounders

## Abstract

Genomic evaluations in pigs could benefit from using multi-line data along with whole-genome sequencing (WGS) if the data are large enough to represent the variability across populations. The objective of this study was to investigate strategies to combine large-scale data from different terminal pig lines in a multi-line genomic evaluation (MLE) through single-step GBLUP (ssGBLUP) models while including variants preselected from whole-genome sequence (WGS) data. We investigated single-line and multi-line evaluations for five traits recorded in three terminal lines. The number of sequenced animals in each line ranged from 731 to 1,865, with 60k to 104k imputed to WGS. Unknown parent groups (UPG) and metafounders (MF) were explored to account for genetic differences among the lines and improve the compatibility between pedigree and genomic relationships in the MLE. Sequence variants were preselected based on multi-line genome-wide association studies (GWAS) or linkage disequilibrium (LD) pruning. These preselected variant sets were used for ssGBLUP predictions without and with weights from BayesR, and the performances were compared to that of a commercial porcine single-nucleotide polymorphisms (SNP) chip. Using UPG and MF in MLE showed small to no gain in prediction accuracy (up to 0.02), depending on the lines and traits, compared to the single-line genomic evaluation (SLE). Likewise, adding selected variants from the GWAS to the commercial SNP chip resulted in a maximum increase of 0.02 in the prediction accuracy, only for average daily feed intake in the most numerous lines. In addition, no benefits were observed when using preselected sequence variants in multi-line genomic predictions. Weights from BayesR did not help improve the performance of ssGBLUP. This study revealed limited benefits of using preselected whole-genome sequence variants for multi-line genomic predictions, even when tens of thousands of animals had imputed sequence data. Correctly accounting for line differences with UPG or MF in MLE is essential to obtain predictions similar to SLE; however, the only observed benefit of an MLE is to have comparable predictions across lines. Further investigation into the amount of data and novel methods to preselect whole-genome causative variants in combined populations would be of significant interest.

## 1 Introduction

Genomic evaluations have been successfully implemented in pig breeding programs to increase the accuracy of predicting genomic EBV (GEBV) and better identify the best animals to be parents of the next generation. However, most evaluations are single-line, or at most, include phenotypes from crossbred progeny to help better evaluate purebred parents. Combining multiple lines could be a possible strategy to allow the comparison of animals across lines and the identification of the best gene combinations (VanRaden et al., 2007). Additionally, small lines can benefit from the increased reference size to have higher GEBV accuracy. Several studies have investigated the impact of combining multiple lines or breeds in farm animals, such as dairy and beef cattle, chicken, and pigs (Calus et al., 2014; Rolf et al., 2015; Song et al., 2017; Cesarani et al., 2022). However, multi-line genomic evaluation (MLE) in pigs is still challenging because the main breeding objective is to improve pure lines for crossbred performance. In contrast, lines have heterogeneous genetic backgrounds and may be distantly related.

Single-step genomic BLUP (ssGBLUP) has been commonly used for genomic evaluation in pigs (Chen et al., 2011; Pocrnic et al., 2019; Song et al., 2019). The fundamental idea of this method is to use all available data, connecting genotyped and non-genotyped animals through a joint relationship matrix **(H)** (Legarra et al., 2009; Christensen and Lund, 2010). For this, ssGBLUP relies on the compatibility between pedigree **(A)** and genomic **(G)** relationship matrices (Misztal et al., 2013). Two significant causes of incompatibility between **A** and **G** are missing pedigrees and heterogeneous base populations (Vitezica et al., 2011; Misztal et al., 2013). In theory, allele frequencies to construct **G** would correspond to the ones from the base population in the pedigree (VanRaden, 2008); however, base animals are seldom genotyped, making base allele frequencies unknown (Legarra et al., 2015). The incompatibility issue becomes critical in multi-line populations because of the heterogeneous base population across lines. Macedo et al. (2020) reported that better genomic predictions could be obtained for such scenarios if the differences in base populations are correctly modeled. Unknown parent groups (UPG) could mitigate this issue by modeling the differences in the genetic base across classes of missing parents and accounting for the differences among breeds or lines; however, UPG assume that the base populations are unrelated (Legarra et al., 2007). Legarra et al. (2015) proposed using metafounders (MF), which are pseudo-animals that act as proxies for the base individuals and can be related. A few studies investigated using ssGBLUP with MF in pigs, but only for crossbred (Xiang et al., 2017; van Grevenhof et al., 2019) and single-line (Fu et al., 2021) evaluations.

Another factor affecting the performance of genomic predictions in MLE is the inconsistent linkage disequilibrium (LD) structure between single-nucleotide polymorphisms (SNP) and quantitative trait loci (QTL) across lines. Pig populations have a smaller effective size (*N*_*e*_) than dairy and beef cattle, resulting in smaller numbers of independent chromosome segments (*M*_*e*_) (Pocrnic et al., 2016b). Therefore, if the lines are distantly related, they are not likely to share many chromosome segments. This could lead to no benefits from combining multiple lines in genomic predictions. Song et al. (2017) evaluated genomic predictions for growth and reproductive traits in pigs using a combined dataset with genotypes for 80k SNP, but no benefits were observed over the single-line genomic evaluation (SLE). In another study, the same authors (Song et al., 2019) showed that pruned whole-genome sequence (WGS) data outperformed the 80k SNP chip for genomic predictions in combined populations; however, no benefit was observed through the direct use of WGS data. This could be due to the redundancy of many SNP across the whole genome with strong LD extent to each other in certain genomic blocks. Therefore, the preselection of significant SNP or removal of redundant SNP could be a possible strategy to improve the accuracy of genomic predictions when WGS data are used. In the case of MLE, a joint preselection of SNP from WGS can help identify variants segregating across lines, which may not be possible with commercial SNP chips because of the limited number of SNP (∼40k to 80k).

In the current study, we aimed to 1) investigate strategies to combine different lines in a multi-line evaluation through the use of unknown parent groups or metafounders and 2) evaluate the impact of using jointly preselected SNP from WGS in multi-line evaluations under ssGBLUP without and with weights from BayesR (Erbe et al., 2012).

## 2 Materials and methods

### 2.1 Data

All datasets were provided by Pig Improvement Company (PIC; Hendersonville, TN). We investigated the average daily feed intake (ADFI), average daily gain (ADG), backfat thickness (BF), ADG recorded in crossbred animals (ADGX), and BF recorded in crossbred animals (BFX) in three terminal pig lines named TL1, TL2, and TL3. These three terminal lines were chosen from the seven lines analyzed in a previous study by Jang et al. (2022a) and Ros-Freixedes et al. (2022). These three lines were chosen because of their large numerical size, completeness of pedigree, and availability of phenotypic data for terminal traits. A two-trait model was considered for the ADFI and ADG (ADFI model), whereas a four-trait model was used for ADG, BF, ADGX, and BFX (GROWTH model). Since ADG and BF were measured in both purebred and crossbred animals (ADGX and BFX), we grouped them together in the four-trait model. Two scenarios were considered in this study: SLE and MLE. The same data were considered for SLE in each line as described in Jang et al. (2022a). For the MLE scenario, all the data from every single line were combined. The total number of records and animals in the pedigree for each line and MLE are given in Table 1. General statistics of the analyzed five traits are given in Table 2. Individuals in each line were genotyped with either GGP-Porcine LD or HD BeadChip (GeneSeek, Lincoln, NE) and jointly imputed for MLE. We filtered out the monomorphic SNP and SNP with a call rate lower than 0.90, minor allele frequency lower than 0.01, and the difference between observed and expected genotype frequencies greater than 0.15. Genotyped pigs with more than 10% missing genotypes were removed as well. For MLE, all genotyped individuals in the three terminal lines were combined. Identical quality control was applied to the imputed MLE chip data (Chip). The total number of genotyped animals in all lines and SNP after quality control is given in Table 3.

**TABLE 1:** Number of records and animals in the pedigree for single- and multi-line datasets.

Lines	ADFI	ADG	BF	ADGX	BFX	Number of animals in pedigree
TL1	35k	0.36M	0.34M	150k	149k	1.13M
TL2	40k	0.30M	0.30M	158k	156k	0.84M
TL3	64k	0.94M	0.86M	299k	247k	3.14M
MLE	140k	1.60M	1.50M	578k	525k	5.28M (5.17M)

^a^ The number of animals in the pedigree for the ADFI model is shown within brackets.

^b^ ADFI: average daily feed intake; ADG: average daily gain; BF: backfat thickness; ADGX: ADG recorded in crossbred; BFX: BF recorded in crossbred.

^c^ TL1: terminal line 1; TL2: terminal line 2; TL3: terminal line 3; MLE: multi-line evaluation.

**TABLE 2:** General statistics for the five traits.

	TL1				TL2				TL3				MLE			
	Min	Max	Mean	SD	Min	Max	Mean	SD	Min	Max	Mean	SD	Min	Max	Mean	SD
ADFI (g/d)	1.10	3.50	2.17	0.30	1.10	3.50	2.03	0.29	1.10	3.49	2.15	0.35	1.10	3.50	2.12	0.33
ADG (g/d)	417	1,123.90	686.84	87.75	423.20	1,092.1	694.94	82.47	450	1,149.4	697	97.46	417	1,149.4	694.32	92.76
BF (mm)	4.80	35	9.98	2.79	5	36.70	9.68	2.75	5	39.70	9.39	2.78	4.80	39.70	9.58	2.78
ADGX (g/d)	302	814	535.20	59.32	300	839	520.19	59.94	300	885	534.12	68.15	300	885	530.68	64.47
BFX (mm)	4.50	49.50	16.39	4.28	4.50	48.00	15.28	3.98	4.50	78.70	16.06	4.90	4.50	78.70	16.01	4.55

^a^ ADFI: average daily feed intake; ADG: average daily gain; BF: backfat thickness; ADGX: ADG recorded in crossbred; BFX: BF recorded in crossbred.

^b^ TL1: terminal line 1; TL2: terminal line 2; TL3: terminal line 3; MLE: multi-line evaluation.

^c^ Min: minimum value in each trait; Max; maximum value in each trait; Mean: mean value in each trait; SD: standard deviation in each trait.

**TABLE 3:** Number of genotyped individuals, SNP, and sequenced animals in single- and multi-line datasets.

Lines	Number of genotyped individuals	Number of SNP	Number of sequenced individuals	Number of imputed sequenced individuals
TL1	60,450	37,909	731	60,474
TL2	41,561	42,897	760	41,573
TL3	104,622	44,022	1,865	104,661
MLE	206,634	41,303	3,356	206,708

^a^ All genotyped individuals were imputed to sequencing using the sequence individuals as the reference.

^b^ TL1: terminal line 1; TL2: terminal line 2; TL3: terminal line 3; MLE: multi-line evaluation.

### 2.2 Whole-genome sequencing and imputation

The WGS data used in this study were generated by Ros-Freixedes et al. (2020), Ros-Freixedes et al. (2022). In brief, a low-coverage sequencing strategy was followed by joint calling, phasing, and imputing the whole-genome genotypes using the “hybrid peeling” method implemented in AlphaPeel (Whalen et al., 2018). The “hybrid peeling” method used all marker array and WGS data that were available across the pedigrees. Imputation was carried out separately in each line. Individuals with low predicted imputation accuracy were excluded, as described by Ros-Freixedes et al. (2020). A total of 60,474 (TL1), 41,573 (TL2), and 104,661 (TL3) WGS individuals remained in each line after quality control. The number of WGS and imputed sequenced individuals for each line is given in Table 3. These individuals were predicted to have an average dosage correlation of 0.97 (median: 0.98), as described in Ros-Freixedes et al. (2022). SNP with a minor allele frequency lower than 0.023 were removed since their estimated dosage correlations were lower than 0.90 (Ros-Freixedes et al., 2020). To account for the information from the relatives in genotype calling and reduce the uncertainty from low coverage, genotypes were called after imputation even for the pigs that were directly sequenced.

### 2.3 Training and test sets

Before the SNP preselection, all animals with (imputed) WGS data were split into two non-overlapping datasets: training and test by line. Test sets were generated by extracting genotyped individuals in the litters from the last generation of the pedigree to resemble the selection candidate evaluation performed by pig breeding companies. Only litters with a minimum of five full-sibs were considered as a way to balance the representation of different families in the test sets, and all remaining individuals were considered training sets. The training sets were additionally defined as those individuals with a pedigree relationship coefficient with any individual in the test set that was lower than 0.5 to reduce the relationship between the training and test sets. Therefore, we can avoid the initially high accuracy generated by the close relationship between the two sets. For multi-line scenarios, the training set was formed by merging the training sets that had been defined for each line. Training datasets were included in the discovery sets for multi-line GWAS in this study. Although using the same sets for training and GWAS can reduce accuracy and increase the bias of genomic predictions (Veerkamp et al., 2016; MacLeod et al., 2017), Ros-Freixedes et al. (2022) observed no systematic changes in the accuracy after splitting the training set into two exclusive subsets, one for the GWAS and one for genomic prediction, using same datasets as in the current study.

### 2.4 Preselected SNP panels

Five different preselected SNP panels were created from WGS for genomic predictions. Two of them were as described in Jang et al. (2022a); Ros-Freixedes et al. (2022): 1) Top40k and 2) ChipPlusSign. Three additional preselected panels were designed for the current study: 3) TopSign, 4) LDTags, and 5) AllComb. Preselection of variant sets (1) to (3) was performed following a multi-line GWAS as described in Ros-Freixedes et al. (2022), which encompassed the training sets of TL1, TL2, TL3, another terminal line, and three maternal lines. Top40k included the variants with the lowest *p*-value (not necessarily below the significance threshold) in each consecutive non-overlapping 55-kb window along the genome, based on multi-line GWAS analyses, so that a similar number of variants than in Chip was retained. TopSign only included significant variants (*p* ≤ 10^−6^), where the significance threshold is based on a significance level of 0.05, accounting for multiple testing through the Bonferroni correction, assuming that markers from Chip were independent, from the multi-line GWAS in a way that when a 55-kb window contained more than one significant variant, only that with the lowest *p*-value was selected. ChipPlusSign combined TopSign and Chip because sometimes the number of significant variants was too small, and empirical results have shown that augmenting marker arrays with WGS variants can be a successful strategy to improve prediction accuracy (VanRaden et al., 2017; Lopez et al., 2021). The LDTags were tag variants retained after pruning, based on LD with an r^2^ > 0.1. This very stringent threshold was adopted to remove many SNP. AllComb contained variants from LDTags, Top40k, TopSign, and Chip. The preselected variant sets (1) to (3) were trait-specific, and the preselected variants for the traits included in each of the ADFI and GROWTH models were combined for our analyses. Thus, in the ADFI model, preselected variants for ADFI and ADG were combined and used for the genomic predictions. Likewise, preselected variants from ADG and BF were merged and used for genomic predictions in the GROWTH model; variants were not selected for ADGX and BFX because crossbred animals were not sequenced. For a fair comparison to the commercial SNP chip data (i.e., Chip), 206,634 animals were extracted from each preselected variant set. Afterward, quality control was performed with the same aforementioned criteria, and those individuals with parent–progeny conflicts were also removed. Table 4 depicts the number of genotyped animals and SNP for all preselected SNP panels after quality control.

**TABLE 4:** Number of animals and SNP in all preselected panels used for multi-line evaluations.

	Number of genotyped animals		Number of SNP	
SNP panels	ADFI	GROWTH	ADFI	GROWTH
Chip	206,634	206,634	41,303	41,303
ChipPlusSign	206,452	206,630	62,906	59,756
LDTags	202,891	202,891	105,720	105,720
AllComb	205,729	205,680	215,361	210,619
Top40k	206,232	206,238	51,297	49,738
TopSign	206,228	206,228	21,772	18,593

^a^ ADFI: two-trait ADFI model (ADFI and ADG).

^b^ GROWTH: Four-trait GROWTH model (ADG, BF, ADGX, and BFX).

^c^ Chip: imputed chip data; ChipPlusSign: preselected SNP panels combining TopSign with Chip; LDTags: preselected SNP panels after LD pruning; AllComb: preselected SNP panels combining Chip, LDTags, Top40k, and TopSign; Top40k: preselected SNP panels consisting the variants with the lowest *p*-value in each 40k window; TopSign: preselected SNP panels consisting of only significant variants.

### 2.5 Single-line genomic prediction

To compare the performance of genomic prediction using single-line and multi-line evaluation, Chip data were tested for SLE. Multi-trait linear mixed models were used to perform genomic predictions with two and four traits for the ADFI and GROWTH models, respectively. Only a four-trait GROWTH model (ADG, BF, ADGX, and BFX) of TL1, TL2, and TL3 is described here:

y=Xb+Wc+Zu+e,

where **y** is the vector of phenotypes; **b** is a vector of fixed effects; **c** is a vector of random litter effects; **u** is a vector of random additive genetic effects; and **e** is a vector of residual effects. Matrix **X** is an incidence matrix relating phenotypes in vector **y** to the fixed effects (contemporary group as a fixed effect for all traits, off-test weight (weight measured at about 140 days of age), and carcass weight as a covariate only for BF and BFX, respectively) in vector **b;** matrix **W** is an incidence matrix for random litter effects in vector **c;** and matrix **Z** is an incidence matrix for random additive genetic effects in vector **u**.

### 2.6 Multi-line genomic prediction with unknown parent groups and metafounders

Multi-trait linear mixed models were used to carry out genomic predictions with two and four traits for the ADFI and GROWTH models, respectively. Two UPG methods or MF were used to model the heterogeneous base across the lines. Herein, only the four-trait GROWTH model (ADG, BF, ADGX, and BFX) is described:

y=Xb+Wc+Zu+ZQg+e,

where **Q** is an incidence matrix relating animals in vector **u** to the UPG in vector **g**. All other matrices and vectors were already described.

The genomic predictions were performed with ssGBLUP without and with weights from BayesR (WssGBLUP) using the BLUPF90 family of programs (Misztal et al., 2014b). In ssGBLUP and WssGBLUP, the inverse of the realized relationship matrix ( $H^{-1}$ ), which combines pedigree and genomic relationships, is represented as follows (Aguilar et al., 2010):

$$H^{-1}=A^{-1}+\left[\begin{array}{cc} 0 & 0\\ 0 & G^{-1}-A_{22}^{-1} \end{array}\right],$$

where $G^{-1}$ is the inverse of the genomic relationship matrix and $A^{-1}$ and $A_{22}^{-1}$ are the inverses of the pedigree relationship matrix for all and genotyped individuals, respectively. **G** was created with method 1 of VanRaden (2008) as

$$G=\frac{\left(M-2P\right)D{\left(M-2P\right)}^{\prime }}{2\sum_{i=1}^{m}p_{i}\left(1-p_{i}\right)},$$

where M contains genotypes coded as 0, 1, and 2; D is a matrix of weights ( D = **I** in ssGBLUP and D ≠ **I** in WssGBLUP); and P is a matrix in which columns contain observed frequencies of the second allele at a locus $p_{i}$ across the entire genotyped population. To avoid singularity issues, **G** was blended with 5% of $A_{22}$ . The GEBV for UPG models was calculated as follows:

GEBV=Qg+u.

We investigated two ways to fit UPG in ssGBLUP. The first considered UPG in **A**, **A**_22_, and **G** (Misztal et al., 2013) and was called UPG1. $H^{-1}$ with UPG1 is described as follows:

$$H_{UPG1}^{*}=A^{*}+\left[\begin{array}{ccc} 0 & 0 & 0\\ 0 & G^{-1}-A_{22}^{-1} & -\left(G^{-1}-A_{22}^{-1}\right)Q\\ 0 & -Q^{\prime }\left(G^{-1}-A_{22}^{-1}\right) & Q^{\prime }\left(G^{-1}-A_{22}^{-1}\right)Q \end{array}\right],$$

where $A^{*}$ is the inverse of **A** with UPG constructed with the QP transformation (Quaas, 1988). The second model related UPG only to **A** and $A_{22}$ , was called UPG2, and had $H^{-1}$ represented by

$$H_{UPG2}^{*}=A^{*}+\left[\begin{array}{ccc} 0 & 0 & 0\\ 0 & G^{-1}-A_{22}^{-1} & -A_{22}^{-1}Q\\ 0 & -Q^{\prime }A_{22}^{-1} & Q^{\prime }A_{22}^{-1}Q \end{array}\right].$$

Alternatively to the UPG models, we used MF to fit the heterogeneous genetic base across different lines (Legarra et al., 2015). Based on the MF theory, the pedigree relationship matrices are modified to be compatible with **G** centered with allele frequencies of 0.5 ( $G_{0.5}$ ) (Christensen, 2012; Legarra et al., 2015). $H^{-1}$ with MF is described as follows:

$$H^{\Gamma -1}=A^{\Gamma -1}+\left[\begin{array}{ccc} 0 & 0 & 0\\ 0 & G_{05}^{-1}-A_{22}^{\Gamma -1} & 0\\ 0 & 0 & 0 \end{array}\right],$$

where $A^{\Gamma -1}$ and $A_{22}^{\Gamma -1}$ are the altered $A^{-1}$ and $A_{22}^{-1}$ with the parameter Γ , which is a matrix of relationships within and across MF. The Γ matrix was computed using SNP markers under a generalized least square approach (Garcia-Baccino et al., 2017) through the gammaf90 program of the BLUPF90 software suite (Misztal et al., 2014b).

For UPG and MF models, five groups of base animals were defined based on the lines of origin. The first three were assigned to TL1, TL2, and TL3; one was assigned to another terminal line (TL4), and the other represented a crossbred line (CL). TL4 is another important terminal line, and it contributed to generating the commercial crossbred data that were used in this study, so we defined it as one group of base animals. Due to an issue of estimating Γ with all animals far back in the pedigree, animals born before 2000 were removed. Therefore, the total number of pedigreed animals was 5.16M for the GROWTH model and 5.04M for the ADFI model after data truncation. Because of the unequal number of phenotypes between the two models, the total number of pedigreed animals differed. The description of groups of UPG and MF is given in Table 5.

**TABLE 5:** Number of animals related to each unknown parent group or metafounder and Γ value.

			Γ				
MF	Males	Females	TL1	TL2	TL3	TL4	CL
TL1	3,649	2,713	1.03	0.45	0.5	0.67	0.46
TL2	2,083	1,949	0.45	1.26	0.44	0.39	0.42
TL3	7,148	6,135	0.5	0.44	0.62	0.43	0.38
TL4	30,141	29,707	0.67	0.39	0.43	1.06	0.42
CL	37,150	41,741	0.46	0.42	0.38	0.42	0.58

^a^ TL1: terminal line 1; TL2: terminal line 2; TL3: terminal line 3; TL4: terminal line 4; CL: crossbred line.

To efficiently compute $G^{-1}$ without the direct inversion of **G**, the algorithm for proven and young (APY) (Misztal et al., 2014a) was applied to SLE and MLE. The number of core animals in each line corresponded to the number of the largest eigenvalues, explaining 98% of the total variation in **G** constructed with Chip data (Pocrnic et al., 2016a). Therefore, the number of core animals was 3,996, 5,739, and 6,848 for TL1, TL2, and TL3, respectively. The number of core animals for MLE was selected as mentioned previously but after combining all three terminal lines. Therefore, the number of core animals in MLE was 8,574. To fairly select the core animals from each line, we sampled 30% (2,572), 20% (1,715), and 50% (4,287) from TL1, TL2, and TL3, respectively. Those numbers were equivalent to the proportion of genotyped animals in each line for the MLE scenario.

In WssGBLUP, BayesR (Erbe et al., 2012) was used to estimate individual SNP variances, which were considered weights. Each iteration stored individual SNP variances, and posterior SNP variance was calculated as the average variance across all the iterations. Afterward, the weights were re-scaled so that the trace of **D** was equal to the number of SNP. More details about using BayesR weights in WssGBLUP are described in Gualdrón-Duarte et al. (2020). To ensure the quality of results from ssGBLUP, traditional BLUP (BLUP) was also performed for both SLE and MLE.

### 2.7 Validation

The LR validation method (Legarra and Reverter, 2018) was used to evaluate model performance. A total of 5,970 (TL1), 3,750 (TL2), and 11,308 (TL3) youngest genotyped animals in the test sets had their phenotypes removed from the evaluation. In the MLE scenario, the total number of records in the ADFI model was 1,476,644 (TL1), 1,478,431 (TL2), and 1,472,021 (TL3). For the GROWTH model, the number of records was 2,187,538, 2,189,326, and 2,182,916 for TL1, TL2, and TL3, respectively. These will be referred to as the reduced data and will be represented by the subscript *r*. On the other hand, the whole data, with no phenotype truncation, will be represented by the subscript *w*. Under the LR method, the accuracy of GEBV was calculated as $\hat{acc}=\sqrt{\frac{cov\left({\hat{u}}_{w},{\hat{u}}_{r}\right)}{\left(1-\bar{F}\right){\hat{\sigma }}_{u}^{2}}}$ , where $\hat{u}$ is the vector of GEBV; $\bar{F}$ is the average inbreeding coefficient for validation animals; ${\hat{\sigma }}_{u}^{2}$ was additive genetic variance in each model. Bias was calculated as the difference between the mean of GEBV from the reduced and whole datasets, which is $\mu_{w,r}={\bar{\hat{u}}}_{r}-{\bar{\hat{u}}}_{w}$ . Bias was then standardized by the respective additive genetic standard deviation to compare all traits on the same scale, with an expected estimator of 0 if unbiased. Dispersion of GEBV was assessed as the deviation of the regression coefficient (b_1_) from 1, where b_1_ was obtained from the regression of ${\hat{u}}_{w}$ on ${\hat{u}}_{r}$ : ${\hat{u}}_{w}=b_{0}+b_{1}{\hat{u}}_{r}$ . Under the condition of neither over nor under dispersion, the expectation of this estimator would be 1.

## 3 Results

### 3.1 Population structure and metafounders ( Γ )

**FIGURE 1:**
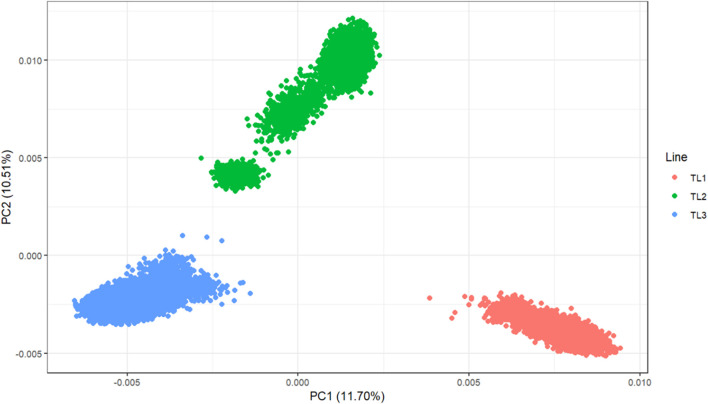
Principal component analysis of the three terminal lines.

Principal component analysis (PCA) was performed to investigate the population structure of the three terminal lines (Figure 1). Chip data were used for 33,714 genotyped animals (TL1: 9,282, TL2: 7,900, and TL3: 16,532). These were selected among 206,634 genotyped animals for efficient computation and had at least one progeny. The PCA plot showed a clear separation among the lines, with the first two principal components explaining 22.1% of the genetic variation. These results reinforce the need to account for different genetic bases when having a multi-line evaluation.

The relationships within and between MF ( Γ ) are given in Table 5. The relationships within the MF (diagonal values of Γ ) were greater than 1 for TL1, TL2, and TL4, indicating that the base populations for those lines are inbred (Legarra et al., 2015). Contrarily, TL3 and CL had values lower than 1, indicating a high frequency of heterozygosity compared to the population mean. All the relationships between the MF (off-diagonal values of Γ ) showed positive values between 0 and 1, suggesting an overlap between ancestral populations.

### 3.2 Accuracy of GEBV

**FIGURE 2:**
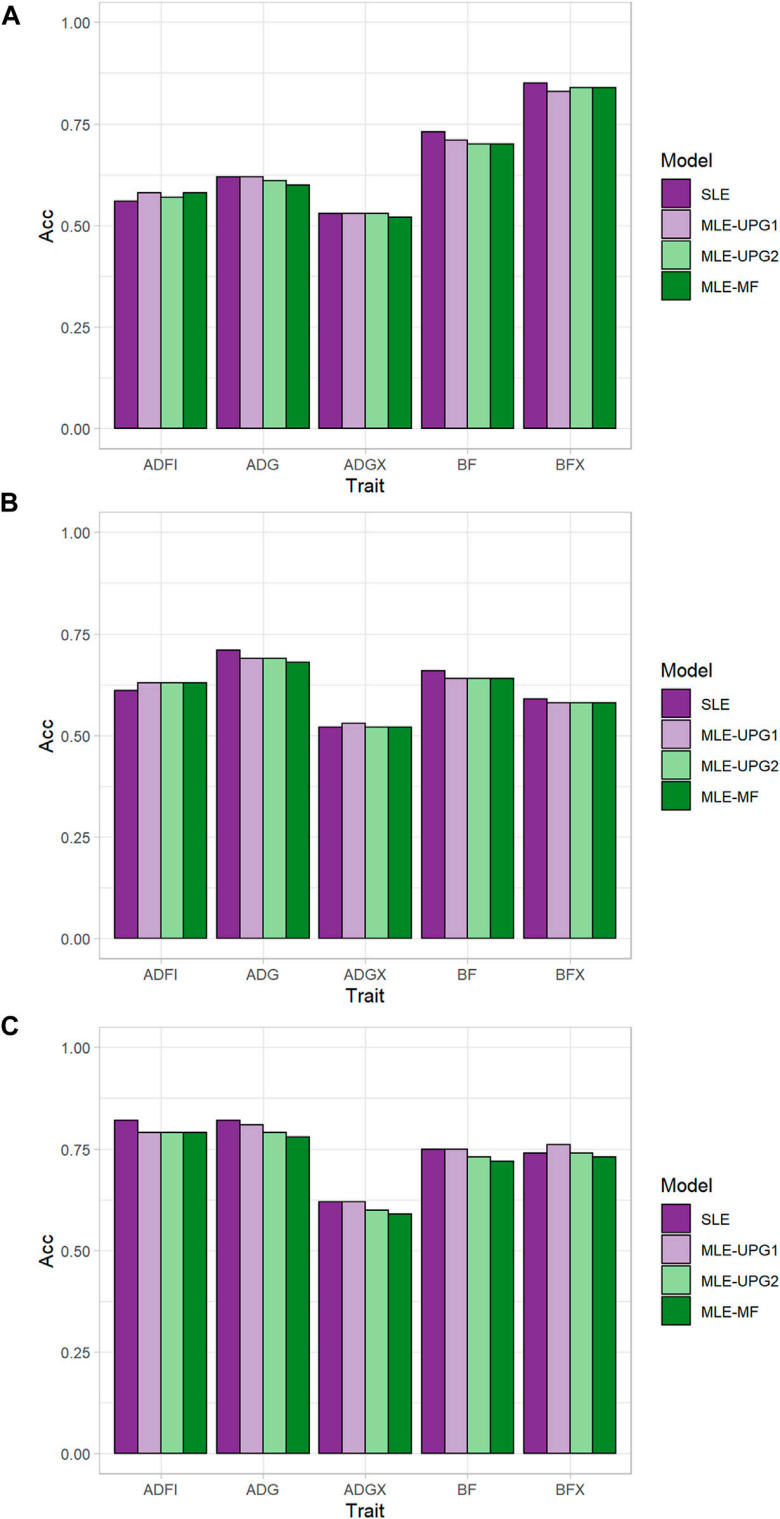
Prediction accuracy of single- and multi-line evaluations with unknown parent groups and metafounders using Chip data for TL1 **(A)**, TL2 **(B)**, and TL3 **(C)**.

Figure 2 shows the accuracy of predicting GEBV in SLE and MLE with UPG1, UPG2, and MF for five traits using Chip data. The difference in prediction accuracy between SLE and MLE was up to 0.04 (Figure 2). The results for TL1 are shown in Figures 2A. Only ADFI showed greater accuracy with MLE than with SLE (0.56) for UPG1 (0.58), UPG2 (0.57), and MF (0.58). On the other hand, SLE had similar or better performance than MLE for the other four traits in the growth model. Among MLE scenarios, UPG1 outperformed UPG2 and MF for many traits, but the differences were minimal. The results for TL2 followed similar patterns (Figures 2B), that is, all MLE scenarios outperformed SLE for ADFI (0.63 vs. 0.61). Additionally, prediction accuracies of MLE scenarios were very similar for TL2. In general, TL3 reported greater prediction accuracies than TL1 and TL2; however, using MLE was not favorable. Only MLE with UPG1 outperformed SLE, which was for BFX (0.76 vs. 0.74). A comparison of MLE scenarios showed that UPG1 performed best for ADG, BF, ADGX, and BFX, with an accuracy gain of up to 0.03 (ADG in TL3).

In the current study, five preselected genotype panels created from WGS were compared to Chip for genomic prediction. As UPG1 showed the best prediction accuracy with Chip among all MLE scenarios, the only results with UPG1 are given in Table 6. The results with UPG2 and MF are given in Supplementary Tables S1, S2, respectively. In the results of TL1, no benefits of using preselected WGS genotype panels were observed, meaning that Chip performed the best. Among WGS preselected panels, ChipPlusSign showed the greatest prediction accuracy for all five traits. Top40k, TopSign, and AllComb had very similar prediction accuracies. However, LDTags displayed the lowest prediction accuracy among all genotype panels. Similar patterns were observed for TL2. In TL3, ChipPlusSign reported the greatest prediction accuracy only for ADFI (0.81). Likewise, for TL1 and TL2, Top40k, TopSign, and AllComb showed very similar results to each other, but lower accuracy was noticed with AllComb. LDTags underperformed compared to the other genotype panels. For comparisons, the results of the accuracy of EBV with traditional BLUP for SLE and MLE are given in Supplementary Table S3.

**TABLE 6:** Prediction accuracy with preselected genotype panels when assigning unknown parent group 1 in the pedigree and genomic relationship matrices.

		Genotype panels					
Lines	Traits	Chip	Top40k	TopSign	ChipPlusSign	LDTags	AllComb
TL1	ADFI	0.58	0.55	0.54	0.57	0.47	0.54
	ADG	0.62	0.58	0.58	0.61	0.48	0.57
	BF	0.71	0.67	0.67	0.70	0.60	0.68
	ADGX	0.53	0.53	0.53	0.53	0.45	0.50
	BFX	0.83	0.74	0.75	0.81	0.66	0.75
TL2	ADFI	0.63	0.55	0.55	0.57	0.44	0.52
	ADG	0.69	0.63	0.64	0.67	0.53	0.61
	BF	0.64	0.60	0.60	0.63	0.51	0.60
	ADGX	0.53	0.48	0.49	0.51	0.39	0.44
	BFX	0.58	0.52	0.53	0.56	0.41	0.49
TL3	ADFI	0.79	0.79	0.79	0.81	0.72	0.78
	ADG	0.81	0.77	0.79	0.81	0.69	0.78
	BF	0.75	0.71	0.73	0.75	0.61	0.71
	ADGX	0.62	0.56	0.58	0.60	0.46	0.53
	BFX	0.76	0.71	0.73	0.74	0.57	0.68

^a^ TL1: terminal line 1; TL2: terminal line 2; TL3: terminal line 3.

^b^ ADFI: average daily feed intake; ADG: average daily gain; BF: backfat thickness; ADGX: ADG recorded in crossbred; BFX: BF recorded in crossbred.

^c^ Chip: imputed chip data; Top40k: preselected SNP panel consisted of the variants with the lowest *p*-value in each 40k window; TopSign: preselected SNP panel consisting of only significant variants; ChipPlusSign: preselected SNP panel combining TopSign with Chip; LDTags: preselected SNP panel after LD pruning; AllComb: preselected SNP panel combining Chip, LDTags, Top40k, and TopSign.

### 3.3 Bias of GEBV

**FIGURE 3:**
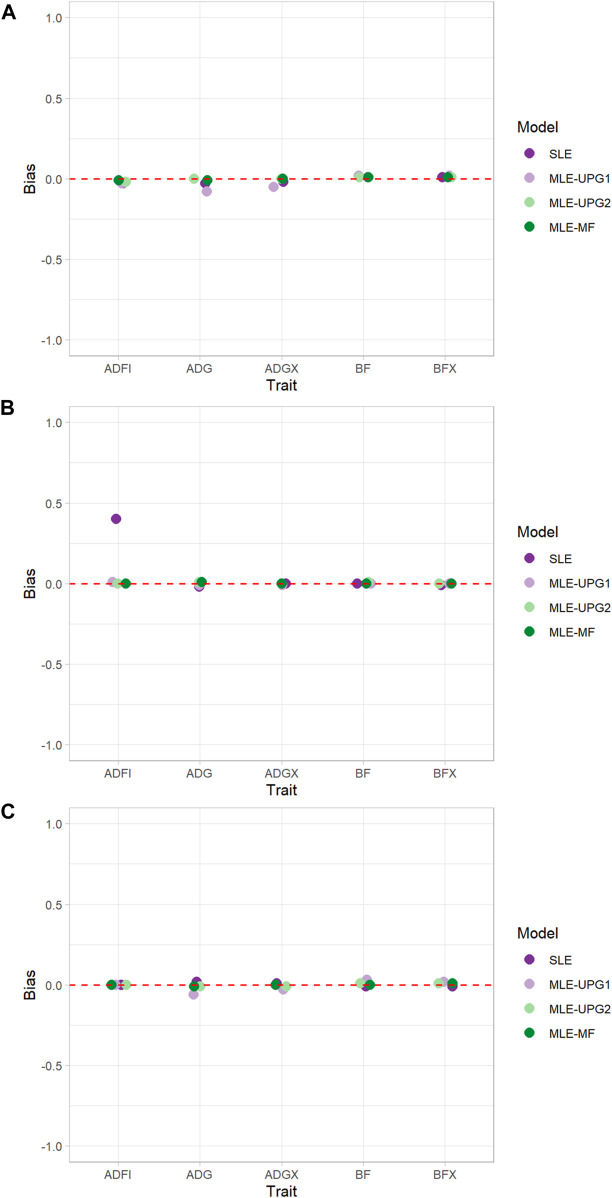
Bias of single- and multi-line evaluations with unknown parent groups and metafounders using Chip data for TL1 **(A)**, TL2 **(B)**, and TL3 **(C)**.

Figure 3 shows the bias of GEBV when using SLE and MLE with UPG1, UPG2, and MF for five traits in each line using Chip data. Overall, almost no bias was observed in all lines and models, except for ADFI with SLE in TL2 (Figures 3B). For TL1, only a slight bias (−0.08–0.02) was displayed for all the traits and models. The maximum bias was for ADG with the MLE-UPG1 model (−0.08). Similarly, TL2 and TL3 also showed a negligible bias for almost all the traits and models, but a large bias of 0.40 for ADFI with the SLE in TL2 was observed.

Bias was also evaluated when the preselected genotype panels from WGS were used for genomic predictions. The results with UPG1 are given in Table 7. Other results with UPG2 and MF are given in Supplementary Tables S4, S5, respectively. For TL1, Top40k indicated less bias for ADFI than Chip. In addition, AllComb showed less bias than Chip for ADG, ADGX, and BFX. Except for these, Chip reported the smallest bias for other traits. Among the preselected panels, Top40k displayed the greatest bias in ADG, BF, ADGX, and BFX. Especially, for ADG and ADGX, the bias was large (−0.58 in ADG and −0.46 in ADGX) with Top40k. ADFI showed almost no bias. Only one case of smaller bias than Chip was identified in TL2 for ADGX with AllComb (0.00). The Chip data showed the smallest bias for the other four traits. AllComb reported the least bias among the WGS preselected panels, whereas other panels indicated inconsistent results varying according to the traits. Interestingly, for TL3, both ChipPlusSign and AllComb reported less bias than Chip for ADG, BF, ADGX, and BFX. In addition, Top40k and TopSign also showed a smaller bias than Chip for ADGX. Contrary to the other WGS preselected genotype panels, bias with LDTags was always greater than with Chip. The results of bias for EBV with traditional BLUP using SLE and MLE are given in Supplementary Table S3.

**TABLE 7:** Bias with preselected genotype panels when assigning unknown parent group 1 in the pedigree and genomic relationship matrices.

		Genotype panels					
Lines	Traits	Chip	Top40k	TopSign	ChipPlusSign	LDTags	AllComb
TL1	ADFI	−0.03	−0.02	−0.03	−0.04	−0.03	−0.03
	ADG	−0.08	−0.58	−0.24	−0.08	−0.15	−0.03
	BF	0.02	0.31	0.13	0.03	0.15	0.05
	ADGX	−0.05	−0.46	−0.18	−0.05	−0.13	−0.02
	BFX	0.02	0.34	0.14	0.03	0.16	0.00
TL2	ADFI	0.01	−0.04	−0.49	−0.02	−0.02	−0.03
	ADG	−0.01	0.17	0.09	0.02	0.04	−0.01
	BF	0.00	0.10	0.06	−0.03	0.13	0.00
	ADGX	−0.01	−0.02	−0.04	−0.05	0.04	0.00
	BFX	0.00	0.03	0.03	−0.03	0.09	0.01
TL3	ADFI	0.00	0.00	0.00	0.00	0.00	0.00
	ADG	−0.06	0.21	0.17	0.00	0.12	0.00
	BF	0.03	0.07	0.06	0.00	0.10	0.01
	ADGX	−0.03	0.01	0.01	0.00	−0.03	0.00
	BFX	0.02	0.02	0.03	0.01	0.05	0.01

^a^ TL1: terminal line 1; TL2: terminal line 2; TL3: terminal line 3.

^b^ ADFI: average daily feed intake; ADG: average daily gain; BF: backfat thickness; ADGX: ADG recorded in crossbred; BFX: BF recorded in crossbred.

^c^ Chip: imputed chip data; Top40k: preselected SNP panel consisting of the variants with the lowest *p*-value in each 40k window; TopSign: preselected SNP panel consisting of only significant variants; ChipPlusSign: preselected SNP panel combining TopSign with Chip; LDTags: preselected SNP panel after LD pruning; AllComb: preselected SNP panel combining Chip, LDTags, Top40k, and TopSign.

### 3.4 Dispersion (inflation/deflation) of GEBV

**FIGURE 4:**
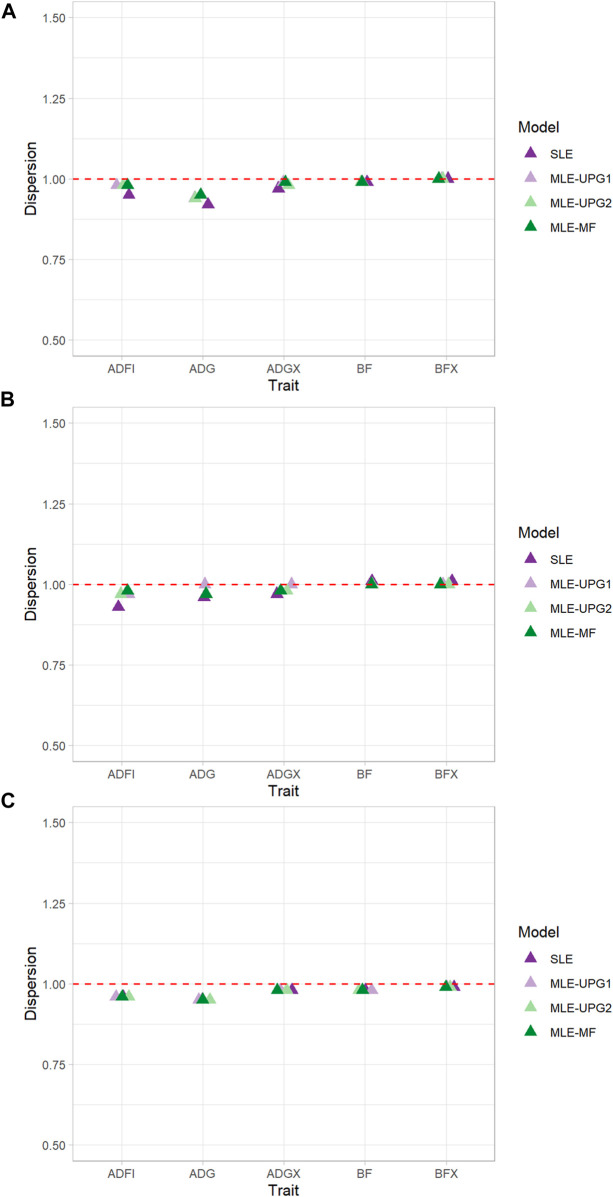
Dispersion (b1) of single- and multi-line evaluations with unknown parent groups and metafounders using Chip data for TL1 **(A)**, TL2 **(B)**, and TL3 **(C)**.

The regression coefficients (b_1_) of GEBV whole on GEBV reduced when using SLE and MLE with UPG1, UPG2, and MF for five traits of each line using Chip data are given in Figure 4. Values of b_1_ greater than 1 indicate deflation of reduced GEBV, and those smaller than 1 indicate inflation. In general, negligible deflation (1.01) and slight inflation (0.92) of GEBV were observed (Figure 4). The results of TL1 indicate relatively greater inflation with SLE for ADFI (0.95) and ADG (0.92) than of other MLE models with UPG and MF, although the differences are minimal. For the other three traits (ADGX, BF, and BFX), all four models reported very similar b_1_ values (0.97–1.00). A similar pattern was identified for ADFI in TL2, which showed the greatest increase of GEBV with SLE (0.93). For the other four traits, no considerable differences were found between the models (0.96–1.01). TL3 reported the most consistent results in each trait. For ADFI, all four models showed b_1_ values equal to 0.96.

To compare the same scenarios with preselected genotype panels from WGS as was performed for prediction accuracy and bias, only the results with UPG1 are given in Table 8. Other results with UPG2 and MF are given in Supplementary Tables S6, S7, respectively. The dispersion of GEBV in TL1 with Top40k (0.94–1.00), TopSign (0.95–1.01), and ChipPlusSign (0.94–0.99) was very close to the result of Chip (0.94–1.00) for all five traits. However, LDTags and AllComb showed greater increase of GEBV than other genotype panels. Among these two, LDTags indicated the greatest increase of GEBV across all the traits (0.77–0.97). Only Top40k and TopSign reported better b_1_ values (close to 1) than Chip for some traits (Top40k–ADFI and ADGX; TopSign–ADFI and ADG). Likewise, Top40k (0.96–1.00), TopSign (0.99–1.01), and ChipPlusSign (0.95–1.00) displayed similar results to Chip (0.97–1.00) in TL2, but a large increase of GEBV with LDTags (0.78–0.90) and AllComb (0.88–0.96). Similar patterns were observed in TL3. Remarkably, TopSign showed less increase of GEBV than Chip for all five traits, although the differences are small (0.02). The results of dispersion for EBV with traditional BLUP using SLE and MLE are given in Supplementary Table S3.

**TABLE 8:** Dispersion (b_1_) with preselected genotype panels when assigning unknown parent group 1 in the pedigree and genomic relationship matrices.

		Genotype panels					
Lines	Traits	Chip	Top40k	TopSign	ChipPlusSign	LDTags	AllComb
TL1	ADFI	0.98	1.00	1.01	0.98	0.84	0.92
	ADG	0.94	0.94	0.95	0.94	0.77	0.88
	BF	0.99	0.99	0.99	0.99	0.91	0.96
	ADGX	0.99	1.00	1.01	0.98	0.91	0.94
	BFX	1.00	1.00	1.01	0.99	0.97	0.98
TL2	ADFI	0.97	0.97	1.00	0.95	0.78	0.88
	ADG	1.00	0.96	0.99	0.97	0.80	0.89
	BF	1.00	1.00	1.01	1.00	0.89	0.96
	ADGX	1.00	0.96	0.99	0.99	0.86	0.92
	BFX	1.00	1.00	1.01	1.00	0.90	0.96
TL3	ADFI	0.96	0.95	0.97	0.96	0.85	0.91
	ADG	0.95	0.94	0.96	0.95	0.81	0.89
	BF	0.98	0.97	0.99	0.98	0.85	0.93
	ADGX	0.98	0.99	1.00	0.97	0.85	0.92
	BFX	0.99	0.98	1.00	0.99	0.90	0.96

^a^ TL1: terminal line 1; TL2: terminal line 2; TL3: terminal line 3.

^b^ ADFI: average daily feed intake; ADG: average daily gain; BF: backfat thickness; ADGX: ADG recorded in crossbred; BFX: BF recorded in crossbred.

^c^ Chip: imputed chip data; Top40k: preselected SNP panel consisting of the variants with the lowest *p*-value in each 40k window; TopSign: preselected SNP panel consisting of only significant variants; ChipPlusSign: preselected SNP panel combining TopSign with Chip; LDTags: preselected SNP panel after LD pruning; AllComb: preselected SNP panel combining Chip, LDTags, Top40k, and TopSign.

### 3.5 Genomic predictions using WssGBLUP

Top40k and ChipPlusSign were used for WssGBLUP with BayesR weighting because these two panels showed the best performance among the preselected genotype panels. Additionally, BayesR weights were considered in ssGBLUP to make the results of this study more comparable to those of Ros-Freixedes et al. (2022), who used the same data under BayesR. The results of prediction accuracy using weights for all five traits are given in Table 9. No benefits of using WssGBLUP over ssGBLUP were observed in Top40k and ChipPlusSign. In fact, the maximum accuracy gain of WssGBLUP compared to ssGBLUP was 0.01; however, a loss in accuracy of up to 0.04 was observed in several traits and lines.

**TABLE 9:** Prediction accuracy of WssGBLUP using BayesR weighting.

Line	Description	ADFI	ADG	BF	ADGX	BFX
TL1	Top40k	0.55	0.58	0.67	0.53	0.74
	Top40k weighted	0.54	0.57	0.67	0.53	0.74
	ChipPlusSign	0.57	0.61	0.70	0.53	0.81
	ChipPlusSign weighted	0.54	0.60	0.69	0.50	0.79
TL2	Top40k	0.55	0.63	0.60	0.48	0.52
	Top40k weighted	0.54	0.62	0.60	0.49	0.52
	ChipPlusSign	0.57	0.67	0.63	0.51	0.56
	ChipPlusSign-weighted	0.53	0.65	0.61	0.47	0.54
TL3	Top40k	0.79	0.77	0.71	0.56	0.70
	Top40k weighted	0.78	0.76	0.71	0.57	0.70
	ChipPlusSign	0.81	0.81	0.75	0.60	0.74
	ChipPlusSign weighted	0.77	0.78	0.72	0.56	0.71

^a^ TL1: terminal line 1; TL2: terminal line 2; TL3: terminal line 3.

^b^ ADFI: average daily feed intake; ADG: average daily gain; BF: backfat thickness; ADGX: ADG recorded in crossbred; BFX: BF recorded in crossbred.

^c^ Top40k: Top40k preselected genotype panel; Top40k weighted: Top40k using BayesR weighting.

^d^ ChipPlusSign: ChipPlusSign preselected genotype panel; ChipPlusSign weighted: ChipPlusSign using BayesR weighting.

## 4 Discussion

In this study, we investigated the impact of using UPG and MF when combining different pig populations in multi-line evaluations. Additionally, we explored the potential benefits of using preselected SNP from WGS in these joint evaluations when SNP received equal or different weights in ssGBLUP. The novel aspect of this study is the amount of sequence data used for multi-line genomic predictions, that is, over 200k pigs. This study provided an insight into how accounting for different genetic bases in three large pig populations could affect the performance of joint genomic evaluations. It also proved that forecasting regarding the usefulness of sequence data for cross-breed predictions does not hold (Meuwissen et al., 2016), at least with the current methods. Overall, three major topics are addressed in this discussion: 1) the impact of fitting UPG or MF in MLE, 2) the usefulness of WGS data for MLE, and 3) the impact of applying different weights to SNP selected from WGS in MLE. In a nutshell, two UPG and one MF models were considered in MLE, and the performances of genomic predictions (accuracy, bias, and dispersion) were compared to those of SLE. Although the results varied depending on the lines and the traits, the maximum changes in prediction accuracy when moving from SLE to MLE were not that large (0.04). In line with the smallest number of genotyped individuals (TL2), all three MLE scenarios performed very similarly. Most of the differences among the scenarios were in the largest line (TL3). Regarding the use of WGS data, almost no benefits were observed when preselected genotype panels were used for genomic prediction compared to Chip. This was true even when different weights were assigned to SNP.

### 4.1 Multi-line genomic evaluation with UPG and MF

Combining populations with different genetic backgrounds in genomic evaluations has been actively investigated in cattle (De Roos et al., 2009; Hayes et al., 2009; Olson et al., 2012; Cesarani et al., 2022), where the primary purpose is to increase the training size for small populations to improve the accuracy of genomic predictions. This is true if there are connections across populations and the training and validation sets are related (Meuwissen et al., 2001; De Roos et al., 2009; Hayes et al., 2009; Zhou et al., 2014). However, combining different pig populations may be challenging even if the lines belong to the same breeding company because the divergence may have happened a long time ago and breeding objectives are different across lines. For these reasons, only a few studies have investigated combining multiple lines, populations, or breeds for genomic predictions in pigs (Fangmann et al., 2015; Aliakbari et al., 2020). The PCA result showed a clear separation among the three lines (representing three different breeds) in our study. As TL2 is the line with the least number of genotyped animals and shows a close distance to TL3 (the largest number), we expected some improvements for TL2 in a joint evaluation. However, only ADFI in TL2 benefitted from MLE instead of SLE, and the increase in accuracy was slight. Additionally, the performance of MF, UPG1, and UPG2 was similar.

In the MF theory, a matrix of relationships within and across metafounders ( Γ ) is used to make pedigree relationships compatible with genomic relationships. We observed relationships within the MF lower than 1 for TL3 and CL but greater than 1 for TL1, TL2, and TL4. Values of Γ smaller than 1 indicate a base population with broad genetic diversity with a higher frequency of heterozygotes relative to the population average (Kluska et al., 2021). On the other hand, a value greater than 1 indicates inbred and related base populations with a lower frequency of heterozygotes relative to the population average (Legarra et al., 2015). In addition, positive Γ values between MF imply overlapping among individuals in the base populations (Kluska et al., 2021). Our results showed only positive Γ values between MF, meaning that there was an overlap between ancestors in their base populations. Xiang et al. (2017) reported similar results using pig data. They calculated Γ values between two MF, which were defined as Landrace and Yorkshire, showing a positive value (0.259). Therefore, we could speculate that although the lines in our study diverged from different breeds, they share ancestors in the base population. In addition, a Γ value smaller than 1 for MF-assigned CL is explained by the fact that this line is crossbred, indicating that a large amount of genetic variability existed in the base population compared to the purebred lines. However, a study by Xiang et al. (2017) reported 0.756 and 0.730 Γ values for Landrace and Yorkshire, respectively, although they were pure breeds. The possible reasons for different Γ values in purebred lines between the current study and the study conducted by Xiang et al. (2017) could be the use of terminal breeds and maternal breeds and different SNP data and lines from different companies.

Fangmann et al. (2015) investigated using multi-subpopulation reference sets to improve the predictive ability of genomic predictions in pigs; however, almost no benefit was reported, even though all the subpopulations diverged from German Large White pigs. Predictive abilities were reduced when distantly related subpopulations were added to the training data. Although our results agree with those of Fangmann et al. (2015), comparing the two studies might be unfair because of the different data sizes and genomic evaluation models. For example, Fangmann et al. (2015) used only 2,053 animals with genotypes and phenotypes under GBLUP. We used ssGBLUP with over 5 M animals, of which 206,634 were genotyped, and 140 k−1.6 M were phenotyped depending on the trait. Most recently, Cesarani et al. [1] performed large-scale multibreed ssGBLUP in dairy cattle using five different breeds with almost 4 M genotyped animals and 29.5 M pedigree records. They reported similar predictive abilities for cows and reliabilities for bulls in single-breed and multibreed evaluations, even though some breeds had less than 50 k genotyped animals and some had more than 500 k. This was attributed to the use of UPG (i.e., UPG2) to model genetic differences across breeds, the inclusion of breed-specific fixed effects in the model, and a fair representation of all the breeds in the APY core. In our study, line-specific fixed effects were modeled to account for nongenetic differences among lines, the APY core properly represented the three lines, and UPG or MF were fit to account for the genetic differences among lines.

A preliminary analysis was performed to compare the performance of genomic predictions in MLE with and without UPG. Most of the traits reported better accuracy, less bias, and less dispersion with UPG1 and UPG2 than the MLE without UPG (results not shown). The major difference between UPG1 and UPG2 was that groups were assigned to **A**, **G**, and $A_{22}$ in UPG1, but only to **A** and $A_{22}$ in UPG2. Theoretically, UPG are not required in **G** because genomic relationships are not affected by missing pedigrees. However, adding UPG to **G** (UPG1) in multi-line evaluations could help adjust the genetic base for each line (Tsuruta et al., 2019). However, UPG1 and UPG2 assume that the base populations are not related. Therefore, MF were also applied to MLE, which considered that individuals in the base populations were related and could be inbred (Legarra et al., 2015). Among the three methods used for MLE, UPG1 had a slight advantage, although the differences between the MLE and SLE models were minimal. Our findings agreed with those obtained by Fangmann et al. (2015), Song et al. (2017), and Aliakbari et al. (2020), which showed that combining lines or breeds had almost no benefits in the performance of genomic predictions compared to the within-line predictions. However, several studies of cattle found some benefits of using the multi-breed reference on genomic prediction (Hayes et al., 2009; Lund et al., 2014). More benefits are likely in populations with a small number of genotyped animals, which was not the case in our study. Although MLE was not advantageous for these pig populations in terms of increased accuracy, having MLE facilitates comparing animals across breeds because of a single base for breeding values. In such a case, having similar accuracy, bias, and dispersion as in SLE is somehow an advantage. It indicates that the lines can be successfully combined to identify “super-boars” to be used across lines if required.

### 4.2 Impact of preselected markers on MLE

In addition to the size of the reference population and the relationships between the reference and test animals (Lourenco et al., 2017; Jang et al., 2022b), another key factor affecting the prediction accuracy is the existence of causal variants or informative variants in LD with them (Wientjes et al., 2015; Veerkamp et al., 2016; Fragomeni et al., 2017). Using multi-line discovery sets could help with the identification of causal variants (or informative variants in LD with them) in MLE settings because the LD between variants may not be consistent across lines, especially for the distantly related ones. Moreover, causative variants for all the lines may not be present in the commercial SNP chips. One plausible approach to help improve genomic predictions in MLE could be using WGS, which possibly harbors all the causal variants. This could increase the power of identifying LD structures and causal variants across lines. However, several studies showed no benefits of using WGS data on genomic prediction without variant preselection (Van Binsbergen et al., 2015; Zhang et al., 2018) because of variant redundancy in WGS. As WGS has highly dense SNP information compared to the regular chip data, variants close to each other would be strongly correlated, providing the same information about nearby QTL. Several studies investigated the impact of variant preselection using WGS data on genomic predictions, showing slight-to-no improvement compared to SNP chips (Brøndum et al., 2015; VanRaden et al., 2017; Fragomeni et al., 2019; Jang et al., 2022b). In addition, several studies in cattle scrutinized the impact of WGS in multi-breed or across-breed genomic predictions (Van Den Berg et al., 2017; Raymond et al., 2018; Meuwissen et al., 2021), but not much is available in pigs (Song et al., 2019; Ros-Freixedes et al., 2022).

Our study used multi-line GWAS or LD pruning to construct preselected genotype panels for different pig terminal lines. Consequently, five genotype sets were designed: ChipPlusSign, Top40k, TopSign, LDTags, and AllComb. Those sets were used for MLE to compare the performance of genomic predictions with Chip. Among all the scenarios, only ChipPlusSign reported greater accuracy than Chip, and this was for one trait (ADFI) only in TL3. On average, ChipPlusSign showed the greatest prediction accuracy among all preselected scenarios but was still smaller than Chip, although the difference was slight. For the multi-line GWAS, we used seven lines (TL1, TL2, TL3, TL4, and three maternal lines) as described in Ros-Freixedes et al. (2022). However, we used only TL1 to TL3 because of the amount of data, completeness of pedigree, and different traits being measured in the terminal and maternal lines. Compared to single-line GWAS, multi-line GWAS could help identify significant SNP affecting the traits as it reduces the long range of LD to short range, allowing more accurate identification of significant SNP across the lines (Moghaddar et al., 2019). This can be especially helpful for species with small *Ne*, such as pigs and chickens with small *Me* and a strong LD extent, which makes the identification of causative variants more difficult (Misztal et al., 2021; Jang et al., 2022b). Several studies in dairy cattle reported up to a 7% increase in reliability when using variants selected from multi-breed GWAS for genomic predictions (Brøndum et al., 2015; van den Berg et al., 2016); however, they use methods other than ssGBLUP. Using ssGBLUP, Fragomeni et al. (2019) reported no benefits of using preselected WGS variants but larger reliabilities than in GBLUP. This is because ssGBLUP allows for more data than in multi-step methods. With enough data, the effects of existing variants are well-captured, and chromosome segments are correctly estimated.

Adding significant SNP to the regular chip data or using only them could potentially improve prediction accuracies only if those are real causative variants with known effects, positions, and variances explained (Fragomeni et al., 2017; Jang et al., 2022b). To accurately identify the significant ones, there should be a sufficient sample size, enough data connected to the genotyped samples, and a robust statistical model, among others. Jang et al. (2022b) extensively investigated the impact of data quantity on the variant selection using simulated WGS and its effect on genomic prediction with populations having different *Ne*. They showed that identifying significant quantitative trait nucleotides (QTN) is more difficult in populations with smaller than larger *Ne* because of the strong LD extent across the genome in the former. Accordingly, improvements in the accuracy of genomic predictions using those selected QTN combined with a 50-k SNP chip in the population with smaller *Ne* were very limited (∼1.98%) compared to the population that had larger *Ne* (∼9.01%). Therefore, although multi-line GWAS could benefit across lines, the benefits would be still limited when it comes to genomic prediction.

### 4.3 Impact of WssGBLUP on MLE

In the current study, we used WssGBLUP with weights computed from BayesR. We did not use the standard weights proposed by Wang et al. (2012) because several studies reported no improvement in the accuracy but increased inflation of genomic predictions when using these weights (Wang et al., 2012; Zhang et al., 2016; Gualdrón-Duarte et al., 2020; Jang et al., 2022b). Additionally, we wanted to make our results comparable to those of Ros-Freixedes et al. (2022), who applied BayesR to the same dataset. In BayesR, SNP effects are sampled from a mixture of four normal distributions with mean zero and variances equivalent to the four classes. Thus, we assumed that better prediction performance could be observed with BayesR weighting if it assigned weights closer to the actual SNP variances; however, no advantages were observed.

This is the first study in large-scale pig lines using MLE with WGS selected variants using the WssGBLUP approach. In practice, the benefits of using WssGBLUP seemed very limited, especially with large datasets and many genotyped animals (Lourenco et al., 2017). Our MLE scenario used around 206 k genotyped animals, and the total number of animals traced back through the combined pedigree data was more than 5 M. In ssGBLUP, any prior information about SNP is overwhelmed by the data because this method allows the use of all sources of information simultaneously, making SNP weighting ineffective (Lourenco et al., 2020).

## 5 Conclusion

This study revealed limited benefits of using preselected whole-genome sequence variants for multi-line genomic predictions, even when hundreds of thousands of animals had imputed sequence data. Correctly accounting for line differences with unknown parent groups or metafounders in multi-line evaluations is essential to obtain predictions similar to single-line evaluations; however, the only observed benefit of a multi-line evaluation is to have comparable predictions across lines. Further investigation into the amount of data and novel methods to preselect whole-genome causative variants in combined populations would be of significant interest.

## Data Availability

The original contributions presented in the study are included in the article/Supplementary Material, further inquiries can be directed to the corresponding author.
