# Supplementary material for: Multi-line ssGBLUP evaluation using preselected markers from whole-genome sequence data in pigs
Source: Front Genet. 2023 May 12;14:1163626. doi: 10.3389/fgene.2023.1163626 (PMC10213539; doi:10.3389/fgene.2023.1163626)
Supplement: Supplementary file 1 [file Table1.docx]

Supplementary Material

Multi-line ssGBLUP evaluation using preselected markers from whole-genome sequence data in pigs

**Sungbong Jang^1*^, Roger Ros-Freixedes^2^, John M. Hickey^3^, Ching-Yi Chen^4^, William O. Herring^4^, Justin Holl^4^, Ignacy Misztal^1^, Daniela Lourenco^1^**

*** Correspondence:** Corresponding Author: jsbng8615@gmail.com

# Supplementary Figures and Tables

For more information on Supplementary Material and for details on the different file types accepted, please see [here](https://www.frontiersin.org/guidelines/author-guidelines#supplementary-material).

## Supplementary table 1. Prediction accuracy with preselected genotype panels when assigning unknown parent groups 2 in the pedigree relationship matrices

|  |  | **Genotype panels** | | | | | |
| --- | --- | --- | --- | --- | --- | --- | --- |
| **Lines** | **Traits** | **Chip** | **Top40k** | **TopSign** | **ChipPlusSign** | **LDTags** | **Allcomb** |
| **TL1** | **ADFI** | 0.57 | 0.59 | 0.53 | 0.56 | 0.47 | 0.54 |
|  | **ADG** | 0.61 | 0.57 | 0.58 | 0.61 | 0.47 | 0.57 |
|  | **BF** | 0.70 | 0.67 | 0.66 | 0.70 | 0.59 | 0.67 |
|  | **ADGX** | 0.53 | 0.53 | 0.54 | 0.53 | 0.45 | 0.50 |
|  | **BFX** | 0.84 | 0.75 | 0.75 | 0.81 | 0.66 | 0.76 |
| **TL2** | **ADFI** | 0.63 | 0.57 | 0.55 | 0.57 | 0.45 | 0.52 |
|  | **ADG** | 0.69 | 0.63 | 0.65 | 0.68 | 0.53 | 0.62 |
|  | **BF** | 0.64 | 0.60 | 0.60 | 0.63 | 0.51 | 0.60 |
|  | **ADGX** | 0.52 | 0.48 | 0.49 | 0.50 | 0.39 | 0.44 |
|  | **BFX** | 0.58 | 0.52 | 0.53 | 0.55 | 0.41 | 0.49 |
| **TL3** | **ADFI** | 0.79 | 0.79 | 0.79 | 0.81 | 0.72 | 0.78 |
|  | **ADG** | 0.79 | 0.77 | 0.79 | 0.81 | 0.69 | 0.78 |
|  | **BF** | 0.73 | 0.71 | 0.73 | 0.75 | 0.61 | 0.71 |
|  | **ADGX** | 0.60 | 0.57 | 0.58 | 0.60 | 0.46 | 0.53 |
|  | **BFX** | 0.74 | 0.70 | 0.73 | 0.74 | 0.57 | 0.67 |

*TL1: Terminal line1; TL2: Terminal line2; TL3: Terminal line3

*ADFI: Average daily feed intake; ADG: Average daily gain; BF: Backfat thickness; ADGX: ADG recorded in crossbred; BFX: BF recorded in crossbred

*Chip: Imputed chip data; Top40k: Preselected SNP panel consisted of the variants with the lowest p-value in each 40k window; TopSign: Preselected SNP panel consisted of only significant variants; ChipPlusSign: Preselected SNP panel combining TopSign to Chip; LDTags: Preselected SNP panel after LD pruning; AllComb: Preselected SNP panel combining Chip, LDTags, Top40k, and TopSign

## Supplementary table 2. Prediction accuracy with preselected genotype panels when assigning metafounders

|  |  | **Genotype panels** | | | | | |
| --- | --- | --- | --- | --- | --- | --- | --- |
| **Lines** | **Traits** | **Chip** | **Top40k** | **TopSign** | **ChipPlusSign** | **LDTags** | **Allcomb** |
| **TL1** | **ADFI** | 0.58 | 0.55 | 0.53 | 0.57 | 0.44 | 0.53 |
|  | **ADG** | 0.60 | 0.54 | 0.55 | 0.59 | 0.42 | 0.53 |
|  | **BF** | 0.70 | 0.65 | 0.64 | 0.69 | 0.56 | 0.66 |
|  | **ADGX** | 0.52 | 0.50 | 0.53 | 0.51 | 0.41 | 0.47 |
|  | **BFX** | 0.84 | 0.72 | 0.74 | 0.81 | 0.64 | 0.74 |
| **TL2** | **ADFI** | 0.63 | 0.51 | 0.51 | 0.55 | 0.39 | 0.50 |
|  | **ADG** | 0.68 | 0.60 | 0.58 | 0.63 | 0.48 | 0.58 |
|  | **BF** | 0.64 | 0.62 | 0.63 | 0.64 | 0.51 | 0.61 |
|  | **ADGX** | 0.52 | 0.47 | 0.47 | 0.48 | 0.35 | 0.42 |
|  | **BFX** | 0.58 | 0.52 | 0.53 | 0.54 | 0.39 | 0.48 |
| **TL3** | **ADFI** | 0.79 | 0.77 | 0.78 | 0.80 | 0.67 | 0.76 |
|  | **ADG** | 0.78 | 0.76 | 0.77 | 0.79 | 0.64 | 0.75 |
|  | **BF** | 0.72 | 0.70 | 0.72 | 0.73 | 0.57 | 0.69 |
|  | **ADGX** | 0.59 | 0.54 | 0.57 | 0.57 | 0.41 | 0.50 |
|  | **BFX** | 0.73 | 0.69 | 0.72 | 0.73 | 0.53 | 0.65 |

*TL1: Terminal line1; TL2: Terminal line2; TL3: Terminal line3

*ADFI: Average daily feed intake; ADG: Average daily gain; BF: Backfat thickness; ADGX: ADG recorded in crossbred; BFX: BF recorded in crossbred

*Chip: Imputed chip data; Top40k: Preselected SNP panel consisted of the variants with the lowest p-value in each 40k window; TopSign: Preselected SNP panel consisted of only significant variants; ChipPlusSign: Preselected SNP panel combining TopSign to Chip; LDTags: Preselected SNP panel after LD pruning; AllComb: Preselected SNP panel combining Chip, LDTags, Top40k, and TopSign

## Supplementary table 3. Prediction accuracy, bias, and dispersion (b1) of traditional BLUP for both single-line and multi-line genomic evaluation

|  |  | **Accuracy** | | **Bias** | | **Dispersion** | |
| --- | --- | --- | --- | --- | --- | --- | --- |
| **Lines** | **Traits** | **SLE** | **MLE** | **SLE** | **MLE** | **SLE** | **MLE** |
| **TL1** | **ADFI** | 0.44 | 0.46 | 0.01 | -0.02 | 0.70 | 0.72 |
|  | **ADG** | 0.43 | 0.46 | -0.03 | 0.00 | 0.64 | 0.66 |
|  | **BF** | 0.59 | 0.59 | 0.00 | 0.01 | 0.85 | 0.84 |
|  | **ADGX** | 0.38 | 0.41 | -0.01 | 0.01 | 0.80 | 0.82 |
|  | **BFX** | 0.72 | 0.75 | 0.03 | 0.01 | 0.94 | 0.94 |
| **TL2** | **ADFI** | 0.42 | 0.48 | -0.01 | 0.01 | 0.72 | 0.75 |
|  | **ADG** | 0.53 | 0.52 | -0.02 | 0.02 | 0.75 | 0.75 |
|  | **BF** | 0.46 | 0.47 | 0.01 | 0.02 | 0.85 | 0.86 |
|  | **ADGX** | 0.40 | 0.41 | -0.01 | 0.02 | 0.82 | 0.83 |
|  | **BFX** | 0.38 | 0.40 | 0.00 | 0.07 | 0.87 | 0.89 |
| **TL3** | **ADFI** | 0.62 | 0.64 | -0.02 | -0.01 | 0.77 | 0.77 |
|  | **ADG** | 0.56 | 0.57 | -0.06 | -0.03 | 0.70 | 0.69 |
|  | **BF** | 0.51 | 0.51 | 0.08 | 0.00 | 0.78 | 0.77 |
|  | **ADGX** | 0.38 | 0.38 | -0.06 | -0.01 | 0.76 | 0.75 |
|  | **BFX** | 0.51 | 0.51 | 0.07 | 0.00 | 0.87 | 0.86 |

*TL1: Terminal line1; TL2: Terminal line2; TL3: Terminal line3

*ADFI: Average daily feed intake; ADG: Average daily gain; BF: Backfat thickness; ADGX: ADG recorded in crossbred; BFX: BF recorded in crossbred

*SLE: Single-line genomic evaluation; MLE: Multi-line genomic evaluation

## Supplementary table 4. Bias with preselected genotype panels when assigning unknown parent groups 2 in the pedigree relationship matrices

|  |  | **Genotype panels** | | | | | |
| --- | --- | --- | --- | --- | --- | --- | --- |
| **Lines** | **Traits** | **Chip** | **Top40k** | **TopSign** | **ChipPlusSign** | **LDTags** | **Allcomb** |
| **TL1** | **ADFI** | -0.02 | -0.04 | -0.03 | 0.40 | -0.02 | -0.03 |
|  | **ADG** | 0.00 | -0.18 | 0.46 | 0.09 | -0.02 | -0.13 |
|  | **BF** | 0.01 | 0.09 | -0.18 | 0.00 | 0.04 | 0.07 |
|  | **ADGX** | 0.00 | -0.12 | 0.32 | 0.04 | -0.02 | -0.08 |
|  | **BFX** | 0.01 | 0.08 | -0.20 | -0.01 | 0.01 | 0.06 |
| **TL2** | **ADFI** | 0.00 | -0.05 | -0.05 | -0.02 | -0.02 | -0.03 |
|  | **ADG** | 0.01 | -0.01 | -0.03 | -0.02 | 0.00 | -0.01 |
|  | **BF** | 0.01 | 0.02 | -0.03 | 0.00 | 0.01 | 0.00 |
|  | **ADGX** | 0.00 | 0.12 | -0.12 | -0.01 | 0.00 | 0.00 |
|  | **BFX** | 0.00 | 0.00 | -0.01 | 0.00 | 0.01 | 0.00 |
| **TL3** | **ADFI** | 0.00 | 0.00 | 0.00 | 0.00 | 0.00 | 0.00 |
|  | **ADG** | -0.01 | 0.11 | -0.32 | -0.01 | 0.09 | 0.00 |
|  | **BF** | 0.01 | 0.02 | -0.04 | 0.00 | 0.04 | 0.01 |
|  | **ADGX** | -0.01 | 0.05 | -0.13 | 0.00 | 0.05 | 0.00 |
|  | **BFX** | 0.01 | -0.01 | 0.05 | 0.01 | 0.01 | 0.01 |

*TL1: Terminal line1; TL2: Terminal line2; TL3: Terminal line3

*ADFI: Average daily feed intake; ADG: Average daily gain; BF: Backfat thickness; ADGX: ADG recorded in crossbred; BFX: BF recorded in crossbred

*Chip: Imputed chip data; Top40k: Preselected SNP panel consisted of the variants with the lowest p-value in each 40k window; TopSign: Preselected SNP panel consisted of only significant variants; ChipPlusSign: Preselected SNP panel combining TopSign to Chip; LDTags: Preselected SNP panel after LD pruning; AllComb: Preselected SNP panel combining Chip, LDTags, Top40k, and TopSign

## Supplementary table 5. Bias with preselected genotype panels when assigning metafounders

|  |  | **Genotype panels** | | | | | |
| --- | --- | --- | --- | --- | --- | --- | --- |
| **Lines** | **Traits** | **Chip** | **Top40k** | **TopSign** | **ChipPlusSign** | **LDTags** | **Allcomb** |
| **TL1** | **ADFI** | -0.01 | -0.03 | 0.00 | -0.01 | -0.02 | -0.13 |
|  | **ADG** | -0.01 | 0.62 | -0.01 | 0.16 | 0.03 | -0.02 |
|  | **BF** | 0.01 | -0.24 | 0.01 | -0.03 | -0.01 | 0.01 |
|  | **ADGX** | 0.00 | 0.43 | 0.00 | 0.11 | 0.04 | -0.01 |
|  | **BFX** | 0.01 | -0.25 | 0.01 | -0.05 | -0.02 | 0.01 |
| **TL2** | **ADFI** | 0.00 | -0.04 | -0.02 | -0.01 | 0.01 | -0.02 |
|  | **ADG** | 0.01 | -0.03 | -0.01 | -0.01 | 0.00 | -0.05 |
|  | **BF** | 0.00 | 0.02 | 0.01 | 0.00 | 0.01 | 0.01 |
|  | **ADGX** | 0.00 | -0.01 | 0.00 | 0.00 | 0.00 | 0.02 |
|  | **BFX** | 0.00 | -0.01 | 0.00 | 0.00 | 0.00 | 0.00 |
| **TL3** | **ADFI** | 0.00 | -0.04 | -0.02 | 0.03 | 0.00 | 0.00 |
|  | **ADG** | -0.01 | -0.17 | -0.01 | -0.01 | 0.01 | 0.05 |
|  | **BF** | 0.00 | -0.04 | 0.00 | 0.00 | 0.00 | 0.01 |
|  | **ADGX** | 0.00 | -0.04 | 0.00 | 0.00 | 0.01 | 0.01 |
|  | **BFX** | 0.01 | 0.04 | 0.00 | 0.01 | 0.01 | 0.00 |

*TL1: Terminal line1; TL2: Terminal line2; TL3: Terminal line3

*ADFI: Average daily feed intake; ADG: Average daily gain; BF: Backfat thickness; ADGX: ADG recorded in crossbred; BFX: BF recorded in crossbred

*Chip: Imputed chip data; Top40k: Preselected SNP panel consisted of the variants with the lowest p-value in each 40k window; TopSign: Preselected SNP panel consisted of only significant variants; ChipPlusSign: Preselected SNP panel combining TopSign to Chip; LDTags: Preselected SNP panel after LD pruning; AllComb: Preselected SNP panel combining Chip, LDTags, Top40k, and TopSign

## Supplementary table 6. Dispersion (b1) with preselected genotype panels when assigning unknown parent groups 2 in the pedigree relationship matrices

|  |  | **Genotype panels** | | | | | |
| --- | --- | --- | --- | --- | --- | --- | --- |
| **Lines** | **Traits** | **Chip** | **Top40k** | **TopSign** | **ChipPlusSign** | **LDTags** | **Allcomb** |
| **TL1** | **ADFI** | 0.98 | 1.00 | 1.00 | 0.97 | 0.84 | 0.91 |
|  | **ADG** | 0.94 | 0.93 | 0.95 | 0.94 | 0.76 | 0.87 |
|  | **BF** | 0.99 | 0.98 | 0.99 | 0.99 | 0.91 | 0.96 |
|  | **ADGX** | 0.98 | 0.97 | 0.99 | 0.99 | 0.89 | 0.94 |
|  | **BFX** | 1.00 | 0.99 | 1.01 | 1.00 | 0.96 | 0.98 |
| **TL2** | **ADFI** | 0.97 | 0.98 | 1.00 | 0.95 | 0.79 | 0.88 |
|  | **ADG** | 0.97 | 0.96 | 0.99 | 0.97 | 0.80 | 0.90 |
|  | **BF** | 1.00 | 1.00 | 1.01 | 1.00 | 0.89 | 0.96 |
|  | **ADGX** | 0.98 | 0.96 | 1.01 | 0.98 | 0.86 | 0.92 |
|  | **BFX** | 1.00 | 0.99 | 1.00 | 1.00 | 0.90 | 0.96 |
| **TL3** | **ADFI** | 0.96 | 0.96 | 0.97 | 0.96 | 0.85 | 0.91 |
|  | **ADG** | 0.95 | 0.94 | 0.96 | 0.95 | 0.80 | 0.89 |
|  | **BF** | 0.98 | 0.97 | 0.99 | 0.98 | 0.85 | 0.93 |
|  | **ADGX** | 0.98 | 0.97 | 0.95 | 0.97 | 0.84 | 0.92 |
|  | **BFX** | 0.99 | 0.98 | 0.99 | 0.99 | 0.90 | 0.96 |

*TL1: Terminal line1; TL2: Terminal line2; TL3: Terminal line3

*ADFI: Average daily feed intake; ADG: Average daily gain; BF: Backfat thickness; ADGX: ADG recorded in crossbred; BFX: BF recorded in crossbred

*Chip: Imputed chip data; Top40k: Preselected SNP panel consisted of the variants with the lowest p-value in each 40k window; TopSign: Preselected SNP panel consisted of only significant variants; ChipPlusSign: Preselected SNP panel combining TopSign to Chip; LDTags: Preselected SNP panel after LD pruning; AllComb: Preselected SNP panel combining Chip, LDTags, Top40k, and TopSign

## Supplementary table 7. Dispersion (b1) with preselected genotype panels when assigning metafounders

|  |  | **Genotype panels** | | | | | |
| --- | --- | --- | --- | --- | --- | --- | --- |
| **Lines** | **Traits** | **Chip** | **Top40k** | **TopSign** | **ChipPlusSign** | **LDTags** | **Allcomb** |
| **TL1** | **ADFI** | 0.98 | 1.00 | 1.01 | 0.99 | 0.91 | 0.96 |
|  | **ADG** | 0.95 | 0.95 | 0.96 | 0.94 | 0.82 | 0.89 |
|  | **BF** | 0.99 | 0.99 | 0.99 | 0.99 | 0.94 | 0.97 |
|  | **ADGX** | 0.99 | 0.92 | 0.99 | 0.97 | 0.92 | 0.95 |
|  | **BFX** | 1.00 | 0.96 | 1.00 | 1.00 | 0.97 | 0.99 |
| **TL2** | **ADFI** | 0.98 | 0.96 | 0.98 | 0.96 | 0.84 | 0.91 |
|  | **ADG** | 0.97 | 0.96 | 0.97 | 0.96 | 0.87 | 0.92 |
|  | **BF** | 1.00 | 1.01 | 1.02 | 1.01 | 0.95 | 0.99 |
|  | **ADGX** | 0.98 | 0.97 | 0.99 | 0.98 | 0.92 | 0.94 |
|  | **BFX** | 1.00 | 1.00 | 1.01 | 1.00 | 0.95 | 0.98 |
| **TL3** | **ADFI** | 0.96 | 0.96 | 0.97 | 0.96 | 0.89 | 0.93 |
|  | **ADG** | 0.95 | 0.95 | 0.96 | 0.95 | 0.86 | 0.91 |
|  | **BF** | 0.98 | 0.98 | 0.99 | 0.98 | 0.90 | 0.95 |
|  | **ADGX** | 0.98 | 0.94 | 0.98 | 0.98 | 0.89 | 0.94 |
|  | **BFX** | 0.99 | 0.99 | 0.99 | 0.99 | 0.94 | 0.97 |

*TL1: Terminal line1; TL2: Terminal line2; TL3: Terminal line3

*ADFI: Average daily feed intake; ADG: Average daily gain; BF: Backfat thickness; ADGX: ADG recorded in crossbred; BFX: BF recorded in crossbred

*Chip: Imputed chip data; Top40k: Preselected SNP panel consisted of the variants with the lowest p-value in each 40k window; TopSign: Preselected SNP panel consisted of only significant variants; ChipPlusSign: Preselected SNP panel combining TopSign to Chip; LDTags: Preselected SNP panel after LD pruning; AllComb: Preselected SNP panel combining Chip, LDTags, Top40k, and TopSign
